# Important role of genetic drift in rapid polygenic adaptation

**DOI:** 10.1002/ece3.5981

**Published:** 2020-01-10

**Authors:** Sona John, Wolfgang Stephan

**Affiliations:** ^1^ Section of Population Genetics Technical University of Munich Freising Germany; ^2^ Leibniz Institute for Evolution and Biodiversity Science Natural History Museum Berlin Germany

**Keywords:** genetic drift, highly polygenic trait, population genetics, rapid adaptation

## Abstract

We analyzed a model to determine the factors that facilitate or limit rapid polygenic adaptation. This model includes population genetic terms of mutation and both directional and stabilizing selection on a highly polygenic trait in a diploid population of finite size. First, we derived the equilibrium distribution of the allele frequencies of the multilocus model by diffusion approximation. This formula describing the equilibrium allele frequencies as a mutation‐selection‐drift balance was examined by computer simulation using parameter values inferred for human height, a well‐studied polygenic trait. Second, assuming that a sudden environmental shift of the fitness optimum occurs while the population is in equilibrium, we analyzed the adaptation of the trait to the new optimum. The speed at which the trait mean approaches the new optimum increases with the equilibrium genetic variance. Thus, large population size and/or large mutation rate may facilitate rapid adaptation. Third, the contribution of an individual locus *i* to polygenic adaptation depends on the compound parameter $\gamma_{i}p_{i}\left(0\right)q_{i}\left(0\right)$, where $\gamma_{i}$ is the effect size, $p_{i}\left(0\right)$ the equilibrium frequency of the trait‐increasing allele of this locus, and $q_{i}\left(0\right)=1-p_{i}\left(0\right)$. Thus, only loci with large values of this parameter contribute coherently to polygenic adaptation. Given that mutation rates are relatively small, this is more likely in large populations, in which the effects of drift are limited.

## Graphical abstract

Rapid adaptation of polygenic traits in a finite size population with optimum shift. We study the effect of standing variation at the mutation–selection–drift equilibrium on adaptation to the new optimum. Random genetic drift plays a critical role in this process and in the detection of this phenomenon at the genome level.

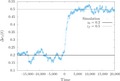

## 1 INTRODUCTION

Adaptation may occur very rapidly in response to changes that are natural or due to human activity. Some recent examples include color variation in guppies (Reznick, 2009), field mice (Vignieri, Larson, & Hoekstra, 2010), and peppered moth (Cook, Grant, Saccheri, & Mallet, 2012); insecticide resistance in Drosophila (Ffrench‐Constant, Bogwitz, Daborne, & Yen, 2002); beak size changes in Darwin's finches (Grant & Grant, 2008); and limb development in Anolis lizards (Losos, 2009). The genetic architecture underlying these phenotypic traits ranges from few genes of major effect (van't Hof, Edmonds, Dalikova, Marec, & Saccheri, 2011) to highly polygenic systems (Lamichhaney et al., 2012, 2015; Linnen et al., 2013).

The analysis of monogenic adaptation, in which one locus in a neutral or weakly selected background is under positive directional selection, has made great progress since the influential work of Maynard Smith and Haigh (1974). Here, a single or very few alleles at selected loci undergo large frequency shifts, possibly sweeping away linked neutral genetic variation—a process called selective sweep. Theoretical studies of selective sweeps have been carried out within the framework of population genetics (reviewed by Jensen, 2014; Stephan, 2019, and others), but these theories do not model the process at the phenotypic level (except for fitness).

On the other hand, polygenic adaptation that involves a large number of selected loci has traditionally been studied using quantitative genetics (Mackay, 2004). Because the quantitative genetic models date back to the time before the genetic mechanisms of inheritance were re‐discovered, they do not refer to the underlying molecular details or dynamics. However, some verbal arguments predict the allele frequencies to change by small amounts when a large number of genetic loci of minor effect sizes control a phenotypic trait (Pritchard & Di Rienzo, 2010). Yet, it is not clear if adaptation can occur rapidly via such subtle changes in the allele frequencies.

There has been a general disconnect between the theories of adaptation that work at either the phenotypic or genotypic level. Ideally, however, one would like to consider models in which selection acts on a phenotypic trait which is connected to the underlying genetics through a genotype‐phenotype map. The response to selection can then be detected at the genetic level and predictions can be made about phenotypic trait evolution. Such a roadmap has been developed by several workers including Bulmer (1972), Barton and Turelli (1989), and Bürger (2000). We follow this direction here to understand the evolutionary dynamics of quantitative traits from the standpoint of population genetics.

We start our investigation from the simple deterministic model that was studied at the equilibrium level by de Vladar and Barton (2014) and whose dynamics after an environmental change was analyzed by Jain and Stephan (2015, 2017a). This model gave some insights into the questions raised above, such as whether and under which conditions rapid adaptation may occur after an environmental shift of the fitness optimum of a phenotypic trait. In these analyses, we have found two distinctly different modes of rapid adaptation: (a) through strong directional selection at a few loci when the effect sizes of the alleles at these loci are large relative to a scaled mutation rate or (b) through weak selection at many individual loci (with small effect sizes) leading to subtle allele frequency shifts in the case of polygenic adaptation. Here, we examine to what extent these deterministic results may be generalized to populations of finite size, in which genetic drift plays an important role. We focus on polygenic adaptation involving a large number of weakly selected loci, since this type of adaptation is not nearly as well studied as the case of strong selection and selective sweeps (with the exception of the very recent work by Simons, Bullaughey, Hudson, and Sella (2018) and Höllinger, Pennings, and Hermisson (2019)). Furthermore, we describe the effect of demography (population size bottlenecks) on polygenic adaptation.

## 2 MODEL

### 2.1 Deterministic model of a single quantitative trait

We consider a single trait that is determined additively (no dominance or epistasis) by *l* unlinked, diallelic loci in a large population of diploids. If the phenotypic effect of the + allele at locus *i* is $\frac{\gamma_{i}}{2}$ and that of the − allele is $\frac{-\gamma_{i}}{2}$, the mean phenotype *c*_1_, the genetic variance *c*_2_ and the skewness *c*_3_ are given by (Jain & Stephan, 2017a)

(1) $$c_{1}=\sum_{i=1}^{l}\gamma_{i}\left(p_{i}-q_{i}\right)=\sum_{i=1}^{l}\gamma_{i}\left(2p_{i}-1\right),$$

(2) $$c_{2}=2\sum_{i=1}^{l}\gamma_{i}^{2}p_{i}q_{i},\;\text{and}$$

(3) $$c_{3}=2\sum_{i=1}^{l}\gamma_{i}^{3}p_{i}q_{i}\left(q_{i}-p_{i}\right),$$

where $p_{i}$ is the frequency of the + allele at locus *i* and $q_{i}=1-p_{i}$ that of the − allele. For simplicity, we assume that the effect‐size distribution is an exponential function with mean $\bar{\gamma }.$ We also assume that the fitness of an individual with trait value *z* has a Gaussian shape centered about the fitness optimum $z_{0}$

(4) $$w\left(z\right)=e^{-\frac{s}{2}{\left(z-z_{0}\right)}^{2}},$$

where *s* measures the strength of selection on the trait. Without loss of generality, we assume $0<z_{0}$ and require that $z_{0}$ $<l\bar{\gamma }.$ The latter condition ensures that the population mean converges to the fitness optimum or to a stationary state close to the optimum (Jain & Stephan, 2015). In a randomly mating population, the change in the allele frequency at the *i*th locus due to selection and mutation is then given by

(5) $$\frac{dp_{i}}{dt}=-s\gamma_{i}p_{i}q_{i}\Delta c_{1}-\frac{s\gamma_{i}^{2}}{2}p_{i}q_{i}\left(q_{i}-p_{i}\right)-\mu p_{i}+\nu q_{i},i=1,\ldots ,l,$$

where $\Delta c_{1}=c_{1}-z_{0}$ is the deviation of the mean phenotype from the fitness optimum. The first term on the right‐hand side of Equation (5) models directional selection toward the phenotypic optimum, the second term describes stabilizing selection in the vicinity of the optimum (Wright, 1935), and the last two terms account for mutation (Barton, 1986; Bulmer, 1972). In agreement with these authors, we assume equal mutation rates μ=ν in our analysis of this model.

### 2.2 Stochastic analysis

To integrate genetic drift into our deterministic model described above, we first consider a diploid population of constant size *N*. We analyze our polygenic model (including drift) under equilibrium conditions based on diffusion theory (Ewens, 2004). However, since this model has a large number of loci, we need to resort to an approximation, which reduces the dimension of the system. Using computer simulations, we then examine the validity of this approximation. Yet, because the number of parameters of our model is relatively large (see above), such simulations are very time‐consuming if the range of biologically relevant parameter values is unknown (as is generally the case for quantitative traits). We therefore chose to examine our approximation using parameter values inferred for the best studied polygenic trait, human height.

Human height is controlled by more than 500 loci (Wood et al., 2014), although recent estimates suggest that this number is probably too high since population structure has not been adequately considered (Berg et al., 2019; Sohail et al. 2019). We choose the following parameter ranges: 0.001–0.01 for the effect sizes (measured in units of the standard deviation, where in the case of human height 1 *SD* ≈ 6.5 cm; Turchin et al., 2012), *s* around 0.1 (Turchin et al., 2012), the number of loci affecting the trait *l* = 200, and mutation rate of about 10^−5^ per generation. The population size is chosen as $N=2\times 10^{4}$, which is close to the long‐term human effective population size. Given these parameter values, we have 2Nμl≫1; thus, the total number of mutations per generation affecting the trait is much larger than 1. Under these conditions, the usual assumption that the phenotypic distribution is well approximated by a normal distribution is justified (Simons et al., 2018). On the other hand, given that the number of mutations per diploid human individual is about 60 per generation (Kong, Frigge, Masson, Besenbacher, & Sulem, 2012), of which less than 10% are functional, suggests that the number of mutations with any functional effect per haploid per generation is less than 3. Therefore, it is plausible that μl≪1. Finally, the chosen values of the effect sizes for most loci fulfill $\gamma_{i}<2\sqrt{\frac{2\mu }{s}}$, which defines the threshold of small‐effect loci under deterministic conditions and symmetric mutation rates (see Results).

### 2.3 Simulations

For the simulations, we first consider a diploid population of constant size *N* to test the assumptions of the stochastic analysis (explained above). In addition, we simulate a demographic model with a major bottleneck resembling the bottleneck inferred from human polymorphism data (Schiffels & Durbin, 2014). The details of the bottleneck model are described in the section on demography.

Stochastic simulations are performed based on a standard Wright–Fisher model (Jain, 2008). We assume that the recombination rate is high and all loci under selection are unlinked. Thus, we calculate the allele frequency changes in each locus independently based on the effect size and the allele frequency of that locus. We start our simulations with all loci having an equal number of + and − alleles. In generation *t* > 0, the allele frequency of the + allele at locus *i* changes by mutation and selection as given by Equation (5). First, we do binomial sampling with mutation based on allele frequency $p_{i}\left(t\right)$. Then, we apply selection by drawing a random number from a binomial distribution whose mean is the modulus of the sum of the two selection terms in Equation (5). This random number is added to or subtracted from the + allele frequency obtained by stochastic sampling (dependent on the sign of the sum of the selection and mutation terms in Equation (5)) to obtain the + allele frequency at locus *i* in the next generation. This process is repeated for all loci for 2*N* generations such that the allele frequencies stabilize.

In the section on the stochastic equilibrium, we run 50,000 independent simulations to obtain the distribution of allele frequencies at each locus. This is compared with the expected steady‐state distribution given by Equation (11). In the adaptation section, we introduce an optimum shift from *z*_0_ to *z_f_* and allow the population to adapt to the new optimum. We calculate the allele frequency trajectory of each locus based on Equation (5) where *z*_0_ is replaced by *z_f_*. Then, we compare the dynamics of the mean deviation from the optimum $\Delta c_{1}\left(t\right)$, the allele frequency trajectories $p_{i}\left(t\right),$ and the allele frequency changes $\delta p_{i}$ (at the end of the short‐term phase) obtained from simulation with Equations ((16), (17), (18)), respectively.

## 3 RESULTS

**Figure 1:**
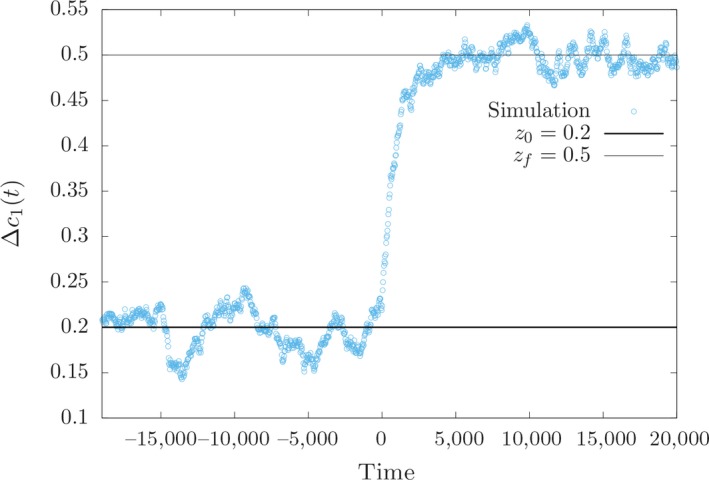
Single run of the trait mean *c*_1_ as a function of time (in generations). The parameter values are: $N=2\times 10^{4}$, *s* = 0.1, *l* = 200, *μ* = 10^−5^ per generation, and $\bar{\gamma }=0.01$. At generation zero the fitness optimum is shifted from *z*_0_ = 0.2 to *z_f_* = 0.5

In Figure 1, we show a typical trajectory of the population mean of the trait obtained by simulation (single run). The population mean fluctuates around an equilibrium state close to the fitness optimum *z*_0_ until—at time zero—it is shifted within a short time span to the new optimum *z_f_*. We consider a diploid population of constant size $N=2\times 10^{4}$. The other parameter values are *s* = 0.1, the number of loci affecting the trait *l* = 200, mutation rate *μ* = 10^−5^ per locus per generation, *z*_0_ = 0.2, *z_f_* = 0.5, and the effect sizes *γ_i_* are drawn from an exponential distribution with mean $\bar{\gamma }=0.01$. In the following, we will analyze these two phases, the equilibrium period before the shift of the fitness optimum and the response of the population after the optimum change.

### 3.1 Stochastic equilibrium between drift, mutation, and selection

As mentioned above, we consider a Wright–Fisher population of *N* diploids, where the population size is assumed to be constant. Thus, the allele frequencies given by Equation (5) may undergo genetic drift, in addition to selection and mutation. We further assume that most of the loci have small effects. In the case of symmetric mutation rates and an infinitely large population size, a precise criterion for this condition can be provided, namely that for most loci $\gamma_{i}$ < $\hat{\gamma },$ where $\hat{\gamma }$ $=2\sqrt{\frac{2\mu }{s}}$(de Vladar & Barton, 2014).

As mentioned above, in equilibrium the mean phenotype *c*_1_ of the population fluctuates around a value close to the fitness optimum *z*_0_ (Figure 1). To analyze this stochastic behavior, we recall that in the deterministic system (polygenic case) the trait mean may change much faster after a perturbation than the allele frequencies (Jain & Stephan, 2015); that is, after the system is pushed away from the stationary state the trait mean may quickly respond, while the allele frequencies reach the stationary state only very slowly. To use this property in our analysis, we write Equation (5) as follows:

(6) $$\frac{d\Delta c_{1}}{dt}=-\frac{s}{2}c_{3}-sc_{2}\Delta c_{1}-2\mu c_{1}$$

and

(7) $$\frac{dp_{i}}{dt}=-s\gamma_{i}p_{i}q_{i}\Delta c_{1}-\frac{s\gamma_{i}^{2}}{2}p_{i}q_{i}\left(q_{i}-p_{i}\right)+\mu \left(q_{i}-p_{i}\right),\;i=1,\ldots ,l-1.$$

Assuming that $\Delta c_{1}$ is a fast variable on the time scale of the allele frequencies $p_{i}$ means that $\Delta c_{1}$ approaches its equilibrium value $\Delta {\tilde{c}}_{1}$ quickly while the allele frequencies need much longer to reach equilibrium (Gardiner, 1990, Chapter 6.4). Under this assumption, we obtain $\Delta {\tilde{c}}_{1}$ by putting the left‐hand side of Equation (6) to zero. Furthermore, we may neglect the skewness term as we focus on loci with small effect sizes $\gamma_{i}$ and $c_{3}$ is proportional to $\gamma_{i}^{3}$ (see Equation (3)). Then, in equilibrium the deviation of the population mean from *z*_0_ is approximately given by

(8) $$\Delta {\tilde{c}}_{1}\approx -\frac{2\mu z_{0}}{s{\tilde{c}}_{2}+2\mu },$$

where ${\tilde{c}}_{2}$ is the equilibrium variance. Thus, for longer times the expected change of the allele frequency $p_{i}$ can be approximated as

(9) $$E\left\{\Delta p_{i}\right\}\approx -s\gamma_{i}p_{i}q_{i}\Delta {\tilde{c}}_{1}-\frac{s\gamma_{i}^{2}}{2}p_{i}q_{i}\left(q_{i}-p_{i}\right)+\mu \left(q_{i}-p_{i}\right),$$

and the variance of the change in $p_{i}$ accounting for the effect of drift is

(10) $$\text{Var}\left\{\Delta p_{i}\right\}\approx \frac{p_{i}q_{i}}{2N}.$$

Using diffusion theory (Ewens, 2004, Chapter 4.5), this leads to the equilibrium frequency distribution of the trait‐increasing allele $p_{i}$ at locus *i*:

(11) $$f\left(p_{i}\right)\approx Cp_{i}^{2\beta -1}q_{i}^{2\beta -1}exp\left(-2\alpha \gamma_{i}\Delta {\tilde{c}}_{1}p_{i}-\alpha \gamma_{i}^{2}p_{i}q_{i}\right),$$

where *C* is the normalization constant (omitting index *i* for locus *i*), α=2Ns, and β=2Nμ is the scaled mutation rate.

Equation (11) has some well‐known properties. If the exponent of the exponential function is very small, such that selection is very weak or the effect sizes are very small (i.e., essentially under the assumption of a mutation‐drift equilibrium), the distribution is U‐shaped when β<0.5, and for larger mutation rates the frequency distribution is rather bell‐shaped. The normalization constant is then given by (Ewens, 2004, Chapter 5.6)

(12) $$C^{-1}\approx B\left(2\beta ,\;2\beta \right)=\frac{\Gamma {\left(2\beta \right)}^{2}}{\Gamma \left(4\beta \right)},$$

where *B* denotes the beta and Γ the gamma function. The mean of this distribution is therefore 0.5, which was also obtained for the deterministic model (de Vladar & Barton, 2014). The variance of the distribution is $1/\left(4\left(4\beta +1\right)\right)$. The standard deviation is therefore large (nearly 0.5) when the scaled mutation rate is small. For large mutation rates, however, the standard deviation is about $1/\left(4\sqrt{\beta }\right)$.

Under the assumption that the exponent of the exponential function is very small (*i.e*., under the assumption of a mutation–drift equilibrium), the genetic variance *c*_2_ at equilibrium can also be calculated in a straightforward way using Equations (11) and (12) in conjunction with Equation (2). We obtain

(13) $${\tilde{c}}_{2}\approx \frac{2\beta }{4\beta +1}\sum_{i=1}^{l}\gamma_{i}^{2}.$$

For exponentially distributed effect sizes with mean $\bar{\gamma }$, the sum on the right‐hand side of Equation (13) may be approximated by $2l{\bar{\gamma }}^{2}$(Jain & Stephan, 2015). Then, the stationary genetic variation is given as

(14) $${\tilde{c}}_{2}\approx \frac{2\beta }{4\beta +1}\sum_{i=1}^{l}\gamma_{i}^{2}\approx \frac{4\beta }{4\beta +1}l{\bar{\gamma }}^{2}.$$

This means that for large mutation rates, the stationary variance converges to $l{\bar{\gamma }}^{2}.$ This result was also obtained for the deterministic model, for which the equilibrium allele frequencies are 0.5. However, for small mutation rates, such that 4β≪1, the stationary genetic variance approaches $4\beta l{\bar{\gamma }}^{2}$, a value that is much smaller than $l{\bar{\gamma }}^{2}$. This has important consequences for the speed of polygenic adaptation, as we will describe below.

Next, we investigate the validity of Equation (11) by simulation. If the exponent of the exponential function of Equation (11) deviates sufficiently from zero, but is still small relative to 1, the normalization constant *C* is not expected to agree with that of the neutral model (given by Equation (12)). Instead, it is approximately given by (see Appendix)

(15) $$C^{-1}\approx B\left(2\beta ,\;2\beta \right)\left[1-\alpha \gamma_{i}\Delta {\tilde{c}}_{1}-\alpha \gamma_{i}^{2}\frac{\beta }{4\beta +1}\right].$$

We also note that in a context where selection cannot be neglected, not only the normalization constant changes, but also the whole shape of the distribution is modified by selection.

**Figure 2:**
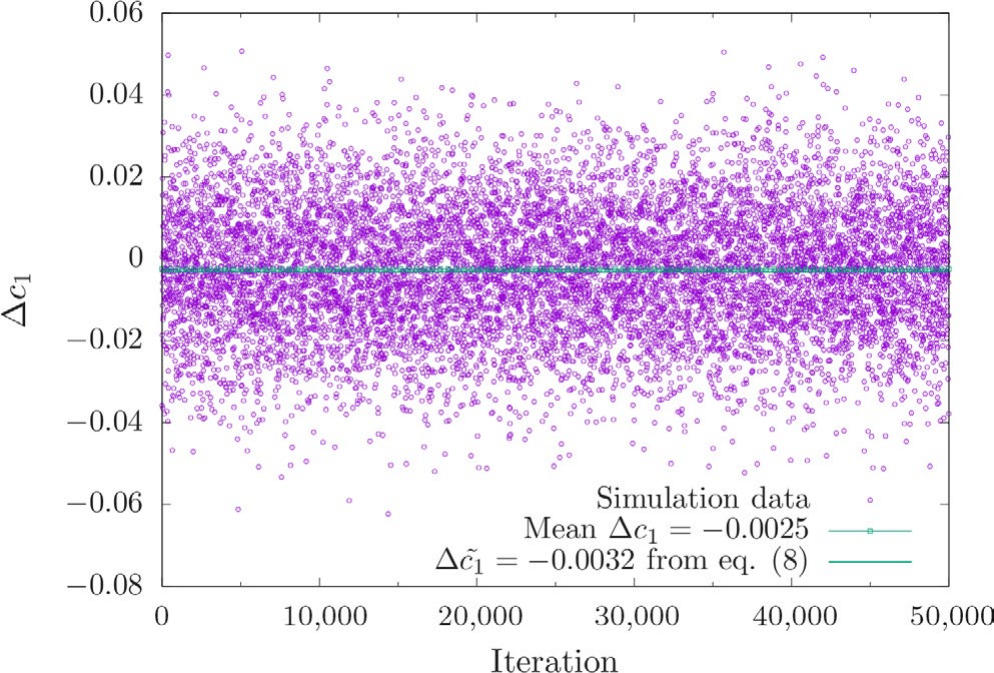
Deviation $\Delta c_{1}$ of the trait mean from the optimum at the time when the fitness optimum changed (see Figure 1). 50,000 simulation runs were performed. The average value of the simulations and the expectation of $\Delta c_{1}$(based on Equation (8)) are shown by a dashed and solid line, respectively

**Figure 3:**
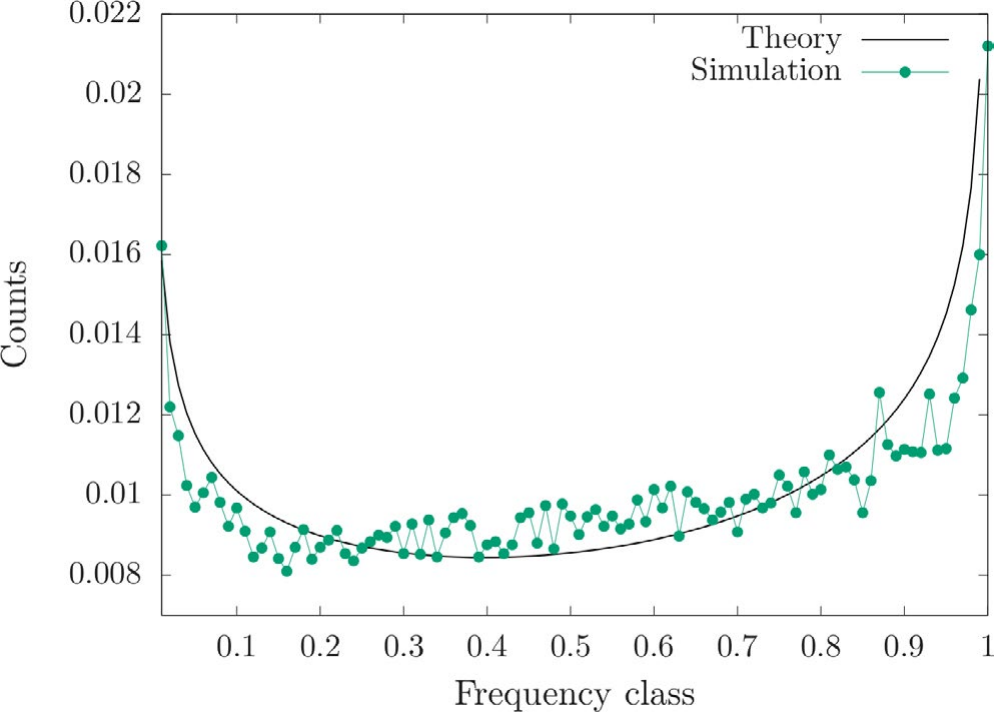
Equilibrium distribution of allele frequencies for a locus with γ = 0.0107 at the time of the environment change. The theoretical curve predicted by Equation (11) is also shown

To examine Equation (11) in conjunction with Equation (15), we simulated our model under the action of drift (for constant population size *N*) and selection for the following set of parameter values: *s* = 0.1, *N* = $2\times 10^{4}$, *l* = 200, *μ* = 10^−5^ $,\bar{\gamma }=0.01$ and *z*_0_ = 0.2. The figures show a reasonable fit of the theoretical predictions with the simulation results averaged over 50,000 independent runs. Figure 2 reveals that the stationary mean deviation is slightly negative as predicted by Equation (8), and Figure 3 compares the simulated frequency distribution with Equation (11) for a particular locus with effect size close to $\bar{\gamma }=0.01$. The stationary second and third moments are shown as Figures S1 and S2, respectively. The simulated variance is somewhat smaller than predicted by Equation (14), which is due to the fact that the latter equation is concerned with the neutral case. The simulated skewness is indeed very small as we have assumed in the derivation of Equation (8).

There are well‐known analytical predictions for the variance of the deviation of the mean phenotype from the optimum. Theories with very different assumptions about mutation (Lande’s (1976) model with no explicit loci, Barton’s (1989) mutation–selection–drift model similar to ours and Sella and Hirsch’s (2005) weak‐mutation Markov chain approximation), all predict that the stationary distribution of the mean deviation from the optimum should have variance 1/(2*Ns*). This is a quite generic property of stochastic processes best known for the Ornstein–Uhlenbeck process (Simons et al., 2018). Indeed, for the values of *N* and *s* used in our simulations we find that based on the above formula the standard deviation of mismatch with the optimum is 0.0158, which is consistent with the results displayed in Figure 2 (where the standard deviation is 0.0160).

### 3.2 Adaptation after a sudden shift of the fitness optimum

Here, we consider a population in which the allele frequency at locus *i*, i=1,…,l, is described by distribution given by Equation (11) when the fitness optimum is suddenly shifted to a new value $z_{f}>z_{0}$, which is also small ($z_{f}<l\bar{\gamma }$). Our goal is to model the dynamics of the alleles at all *i* loci until the population has adapted to the new optimum, that is, until the population mean has reached a value at or close to $z_{f}$ (Figure 1). Describing this dynamics by a multi‐dimensional diffusion equation is very difficult. However, when adaptation after the environmental change is assumed to be fast, we may resort to a deterministic analysis, following that of Jain and Stephan (2017a). This may be justified when the scaled selection coefficient of the + allele at locus *i*, which—immediately after the environmental change—is given by $2Ns\gamma_{i}\left(z_{f}-z_{0}\right)$, is sufficiently large.

Under these assumptions, we get the mean deviation from the new fitness optimum, $\Delta c_{1}=c_{1}-z_{f},$ and the frequencies of the + alleles as (Jain & Stephan, 2015)

(16) $$\Delta c_{1}\left(t\right)\approx \Delta c_{1}\left(0\right)\exp\left(-sc_{2}\left(0\right)t\right)$$

and

(17) $$p_{i}\left(t\right)\approx \frac{p_{i}\left(0\right)}{p_{i}\left(0\right)+q_{i}\left(0\right)\exp\left[\frac{\gamma_{i}\Delta c_{1}\left(0\right)}{c_{2}\left(0\right)}\left(1-e^{-sc_{2}\left(0\right)t}\right)\right]},$$

where the initial condition $p_{i}\left(0\right)$ for each locus is drawn from the stationary distribution given by Equation (11). The time variable *t* is measured such that *t* = 0 is the timepoint when the environment changes.

Derivations of Equation (16) can already be found in the classical literature of quantitative genetics under the assumption that the genetic variance is constant (e.g., see Equations (17) and (18) in Lande (1976)). However, Jain and Stephan (2017a) showed that it can also be derived without this additional assumption. Furthermore, Equation (17) is equivalent to the first formula of equations (24) and (25) in Chevin and Hospital (2008).

Equation (16) defines the short‐term phase of the adaptive process (Jain & Stephan, 2017a). The short‐term phase is defined as the time until the phenotypic mean reaches a value close to the new optimum. During this time, the genetic variance is essentially constant. Depending on the value of the genetic variance, this period, which lasts about ${\left(sc_{2}\left(0\right)\right)}^{-1}$ generations, may be very short when the variance is large. According to Equation (14), this is the case when the number of loci controlling the trait is large and/or the scaled mutation rate *β* is not too small. The role of the mutation rate on the stationary genetic variance, and hence on the speed of polygenic adaptation, was not noticed in our previous deterministic analyses (Jain & Stephan, 2015, 2017a). Here, we find that the genetic variance may be much reduced below the deterministic value of $l{\bar{\gamma }}^{2}$ when the mutation parameter is small such that the distribution given by Equation (11) is extremely U‐shaped. Such a low value of *β* may not lead to rapid adaptation (see Equations (14) and (16)). When *β* is so low such that only a few loci are polymorphic at any time, our model of polygenic selection is no longer applicable.

In the example we use in our simulations mimicking adaptation of human height, the speed of polygenic adaptation is not expected to be much reduced compared to the deterministic case, since several authors found evidence of very recent polygenic adaptation in human height (e.g., Turchin et al., 2012). Indeed, the stationary variance according to Equation (14) is about 0.62 $l{\bar{\gamma }}^{2}$ (further discussed below).

Equation (17) informs us about the frequency shifts $\delta p_{i}$ of the alleles during the short‐term phase. Since we assume that $z_{f}>z_{0}$ and thus $\Delta c_{1}\left(0\right)<0$, the allele frequencies $p_{i}\left(t\right)$ are expected to increase with time at all loci. Indeed, according to Equation (17), the allele frequency shifts at the end of the short‐term phase (i.e,. after ${\left(sc_{2}\left(0\right)\right)}^{-1}$ generations) for sufficiently small effect sizes are approximately

(18) $$\delta p_{i}\approx -\gamma_{i}p_{i}\left(0\right)q_{i}\left(0\right)\Delta c_{1}\left(0\right)\frac{1-e^{-1}}{c_{2}\left(0\right)}.$$

This result suggests that—in the deterministic case—the allele frequency shift at a locus depends strongly on the compound parameter $\gamma_{i}p_{i}\left(0\right)q_{i}\left(0\right).$ Thus, it increases with the effect size and is greatest for initial frequencies around 0.5. Furthermore, Equation (18) shows that after an environmental change the allele frequencies are expected to shift coherently into the same direction. This appears to be an important property of polygenic selection because it may help detecting this type of selection, although the frequency shifts at individual loci are in general small (discussed in Stephan (2016) and Jain and Stephan (2017b)).

**Figure 4:**
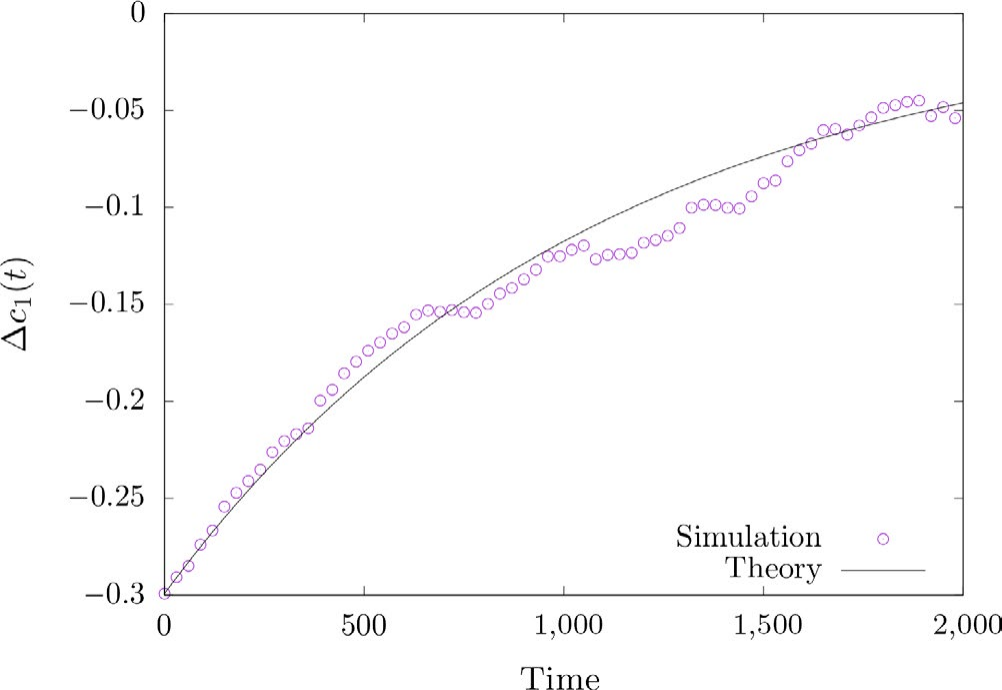
$\Delta c_{1}$ in the short‐term phase after the optimum shift (single run) and comparison with Equation (16)

The stochastic analysis by simulation, however, reveals a more complex picture of polygenic adaptation. First, we find a very good agreement between Equation (16) and the simulation for the deviation $\Delta c_{1}$ of the population mean from the optimum within the short‐term phase, as shown in Figure 4. Second, for the allele frequencies we get a reasonable agreement of Equation (17) with simulations when the effect sizes are sufficiently large and allele frequencies at the time of the environmental shift are around 0.5 (Figure S4). In this case, the allele frequencies increase with time, as predicted by our deterministic analysis. However, the fit is generally poor in Figures S3 and S5, in which the initial allele frequencies are higher or lower than 0.5. The latter figures strongly suggest that besides effect size the initial frequency of the allele frequency plays an important role. Reviewing all three online figures, it appears that the agreement of theory and simulation is best if the allele frequency at the time of the optimum shift is around 0.5.

**Figure 5:**
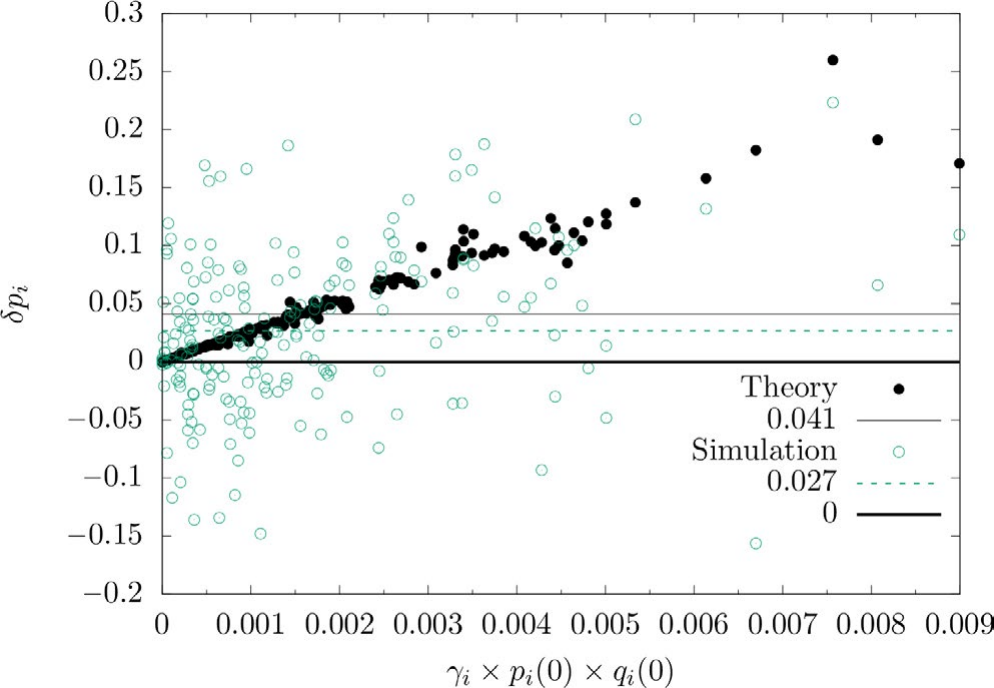
Allele frequency shift $\delta p_{i}$ at locus *i*, *i* = 1, …, 200, in the short‐term phase after the environment change versus the compound parameter $\gamma_{i}p_{i}\left(0\right)q_{i}\left(0\right).$ The filled circles denote the theoretical expectations predicted by the deterministic model (Equation (18)); their average over all 200 loci is shown by the solid line, while the average over the simulated values is indicated by the dashed line

To further explore this issue, we analyzed the differences $\delta p_{i}$ between the simulated allele frequencies at the end of the short‐term phase and those at *t* = 0 for each locus. This shows that—on average—the differences $\delta p_{i}$ are positive as predicted by Equation (18) (paired t‐test *P* = 1.25 × 10^‐8^). However, at many loci negative values are observed. This is clearly seen in Figure 5, in which $\delta p_{i}$ is plotted against the compound parameter $\gamma_{i}p_{i}\left(0\right)q_{i}\left(0\right),$ the critical parameter of the deterministic case. The figure shows that $\delta p_{i}$ is negative for many loci with low values of $\gamma_{i}p_{i}\left(0\right)q_{i}\left(0\right)$, but positive for all loci above a certain threshold. Therefore, the contributions of individual loci to polygenic adaptation depend critically on the parameter $\gamma_{i}p_{i}\left(0\right)q_{i}\left(0\right)$. Figure 5 summarizes our findings, which show that there is a good agreement between theory and simulation only for loci with large $\gamma_{i}$ and/or $p_{i}\left(0\right)$ around 0.5.

### 3.3 Effects of a bottleneck on polygenic adaptation

Here, we assume that population size varies with time. Thus, we are considering the effects of genetic drift combined with demography (varying population size) on polygenic adaptation. Specifically, we simulated a simple demographic model with a major bottleneck resembling the bottleneck inferred from human polymorphism data (Schiffels & Durbin, 2014). The main question we address in this section is whether the genetic variance, which may determine the speed of adaptation of a polygenic trait to a large extent (see Equation (16)), is affected by this bottleneck.

We started our simulation in the past with a population size $N=2\times 10^{4}$. *N* remains constant for several thousand generations (such that the populations reached equilibrium) before it decreased instantaneously to 3,000 individuals. This timepoint mimics the beginning of the human Out‐of‐Africa movement. The population then stayed at this bottleneck size for 5,000 generations before it instantaneously changed back to the constant size of $2\;\times \;10^{4}$. 5,000 generations after this size change we stopped the simulations. The results are as follows. In the pre‐bottleneck phase, *c*_1_ is close to the fitness optimum *z*_0_ = 0.2, such $\Delta c_{1}$ is slightly negative (as in Figure 2). During the bottleneck, *c*_1_ fluctuates greatly, thereby decreasing to an average value such that $\Delta c_{1}$ is almost 60% lower than before the bottleneck. In the third phase, after population size recovered to $2\times 10^{4},$ *c*_1_ increases again slightly, but remains lower than at the beginning of the bottleneck. Thus, due to the bottleneck effect the population mean of the trait deviates from the fitness optimum more than before the bottleneck. This observation is obviously caused by genetic drift. Indeed, drift reduces the genetic variance at the end of the bottleneck phase by about 40% (relative to its value at the beginning of the bottleneck) and may thus have a considerable effect on the speed of adaptation in humans. Furthermore, although Equation (8) describes an equilibrium scenario, this equation may qualitatively explain the larger deviation of the trait mean from the optimum at the end of the bottleneck.

The relatively large fluctuations during the bottleneck (not shown) are probably also due to the increased strength of genetic drift (compared to the initial phase). Drift may cause the system to change between the many deterministic equilibrium points (Barton, 1989). This has been examined in detail for the corresponding two‐locus model of stabilizing selection (Pavlidis, Metzler, & Stephan, 2012; Wollstein & Stephan, 2014): deterministic equilibrium points may be approached, but the trajectories may not stay at the equilibria. Drift may lead to frequent crossings of the separatrices in the phase plane.

## 4 DISCUSSION

### 4.1 Overview

We analyzed a polygenic model formulated explicitly in population genetic terms. This model describes the effects of both directional and stabilizing selection and of mutation on a single quantitative trait in a diploid population of finite size. First, we derived the equilibrium distribution of the allele frequencies by diffusion approximation under the assumption that the trait mean is a fast variable on the time scale of the allele frequencies. This led to a formula describing the equilibrium distribution at each unlinked locus as a mutation–selection–drift balance (Equation (11)). We tested this equation by computer simulation using parameter values inferred for human height, a well‐studied polygenic trait. Second, assuming that a sudden environmental shift of the fitness optimum occurs while the population is in equilibrium, we studied the adaptation of the trait to the new optimum in the short‐term phase (defined by ${\left(sc_{2}\left(0\right)\right)}^{-1}$ generations). The speed of adaptation depends critically on the equilibrium genetic variance, which is approximately constant and given by Equation (14). Thus, the genetic variance of a population with small size and/or low mutation rate may deviate greatly from the deterministic value, namely $l{\bar{\gamma }}^{2}$ (Jain & Stephan, 2015, 2017a). Third, the contribution of an individual locus *i* to polygenic adaptation in the presence of genetic drift depends on the compound locus‐specific parameter $\gamma_{i}p_{i}\left(0\right)q_{i}\left(0\right)$, such that only for large values of this parameter the frequency shift of the trait‐increasing allele at locus *i* in the short‐term phase is coherently positive (i.e., for large effect sizes and/or initial allele frequencies around 0.5). Fourth, we found that population size bottlenecks may keep the trait mean further way from the fitness optimum (than a constant population size) by decreasing the genetic variance of the population. In the following, we discuss the consequences of these findings for polygenic adaptation.

### 4.2 Implications for the detection of polygenic selection

Our results show that the detection of polygenic selection in the genome may be hampered by the effects of genetic drift. Since in the polygenic case selection on individual loci is generally weak, the detection of it is facilitated when the allele frequencies shift in the same direction after an environmental change (Jain & Stephan, 2017b). Such a coordinated shift is predicted by the deterministic model (see Equation (18)). However, in a finite population experiencing drift we found a more complex picture, namely that only for sufficiently large values of the parameter $\gamma_{i}p_{i}\left(0\right)q_{i}\left(0\right)$ the frequency shift of the trait‐increasing allele at locus *i* in the short‐term phase is positive (Figure 5). Thus, depending on the distribution of effects and the allele frequencies at the time when the environment changes, the detection of polygenic selection in the genome may be difficult.

### 4.3 Effects of population size bottlenecks and rapid adaptation in humans

Adaptation of populations to new environments is often accompanied by population size bottlenecks. Because bottlenecks reduce the genetic variance, they may lead to larger deviations of the trait mean from the optimum. This is predicted qualitatively by our Equation (8) (although this equation describes an equilibrium scenario). Furthermore, it was demonstrated by simulation. For our simulations, we used parameter values that mimic the evolution of human height. Our simulations suggest that due to a major bottleneck of about 5,000 generations after humans moved out of Africa the population mean of this trait deviated about 60% more from the phenotypic optimum than before the bottleneck and genetic variation was reduced by about 40% at the end of the bottleneck.

Although genetic drift may reduce the chance to detect polygenic selection in the genome (see above), adaptive differences in human height between southern and northern populations in the past 100 generations have been observed (Turchin et al., 2012). This suggests that the genetic variance was relatively high in the human population before the bottleneck and was not too severely reduced during the bottleneck, as indicated by our simulations. These results appear to be consistent with Equation (14), which predicts a relatively high value of the stationary genetic variance of 0.62 $l{\bar{\gamma }}^{2}$ for a constant population of size *N* = $2\times 10^{4}$ and *μ* = 10^−5^ (see above).

### 4.4 Extension of the model

In our model, we considered only a single trait that is controlled by a large number of loci. Some aspects of the model, however, can also easily be generalized to selection on multiple traits (pleiotropy). For instance, to examine the effect of pleiotropy on the speed of adaptation after a sudden environmental change, we consider the pleiotropic model that was recently proposed by Simons et al. (2018). In this model, an individual's phenotype is described as a vector in an *n*‐dimensional Euclidian space, in which each dimension corresponds to an additive, continuous quantitative trait. The focus is on one of these traits, where the total number of traits parameterizes pleiotropy. Fitness is assumed to decline with distance from the optimal phenotype and is described by a Gaussian distribution.

Then, for a large extent of pleiotropy (large *n* values) the expected changes in the mean traits ${\vec{c}}_{1}$ are given by (Simons et al., 2018, Equation (A46))

(19) $$E\left\{\Delta {\vec{c}}_{1}\right\}\approx -\frac{c_{2}}{w^{2}}\Delta {\vec{c}}_{1},$$

where $c_{2}\ll w^{2}.$ Here, ${\vec{c}}_{1}$ is a vector encompassing the mean values of the traits, $\Delta {\vec{c}}_{1}$ measures the deviations of the population means from the trait optima, $c_{2}$ is the genetic variance of the population as above, and *w* ^2^ quantifies the strength of selection and is given in our model by 1/*s*. In the case n=1, Equation (19) is thus identical to our Equation (6) if mutation and the third moment are neglected.

For the expected change of the allele frequency *p* at the focal locus due to selection Simons et al. (2018) found (see their Equation (A48))

(20) $$E\left\{\Delta p\right\}\approx -\frac{\Delta {\vec{c}}_{1}\cdot \Delta \vec{\gamma }}{w^{2}}pq-\frac{1}{2}\frac{\gamma^{2}}{w^{2}}pq\left(q-p\right).$$

Here, $\gamma^{2}$ is the square of the magnitude of vector $\vec{\gamma }$, which contains the effect sizes of the focal locus on the n traits. The dot denotes a scalar product between $\Delta {\vec{c}}_{1}$ and $\vec{\gamma }$. Therefore, in the case of a single trait Equation (20) agrees with the selection part of Equation (5).

The conclusion from this result is that in the highly pleiotropic case the strength of directional selection depends not only on the effect sizes of the alleles on the traits (summarized in vector $\vec{\gamma }$), but also on the angle between $\Delta {\vec{c}}_{1}$ and $\vec{\gamma }.$ This observation agrees with Lande’s (1979) general results on multivariate selection. If the vectors $\Delta {\vec{c}}_{1}$ and $\vec{\gamma }$ are not parallel, the speed of adaptation is reduced.

## Conflict of Interest

None declared.

## AUTHOR CONTRIBUTIONS

WS and SJ conceived the study. SJ performed the simulations, analyzed and visualized the data. SJ and WS did the analytical work and wrote the manuscript.

## Data Availability

The information needed to perform the simulations is provided in the Model section. Data sharing is not applicable to this article as no new data are analyzed in this study.

## Appendix 1

### Derivation of Equation (15)

We write the normalization constant *C* (omitting index *i* for locus *i*) as

$$C^{-1}=\int_{0}^{1}p^{2\beta -1}q^{2\beta -1}\exp\left[-2\alpha \gamma \Delta {\tilde{c}}_{1}p-\alpha \gamma^{2}pq\right]dp.$$

and expand the exponential function up to the linear term of the Taylor series. This results in a sum of three integrals

$$C^{-1}\approx B\left(2\beta ,2\beta \right)-2\alpha \gamma \Delta {\tilde{c}}_{1}B\left(2\beta +1,2\beta \right)-\alpha \gamma^{2}B\left(2\beta +1,2\beta +1\right),$$

which can be expressed as beta functions. Using well‐known properties of the beta function leads then immediately to Equation (15).
