# Supplementary figures and images for: Important role of genetic drift in rapid polygenic adaptation
Source: Ecol Evol. 2020 Jan 10;10(3):1278–87. doi: 10.1002/ece3.5981 (PMC7029068; doi:10.1002/ece3.5981)

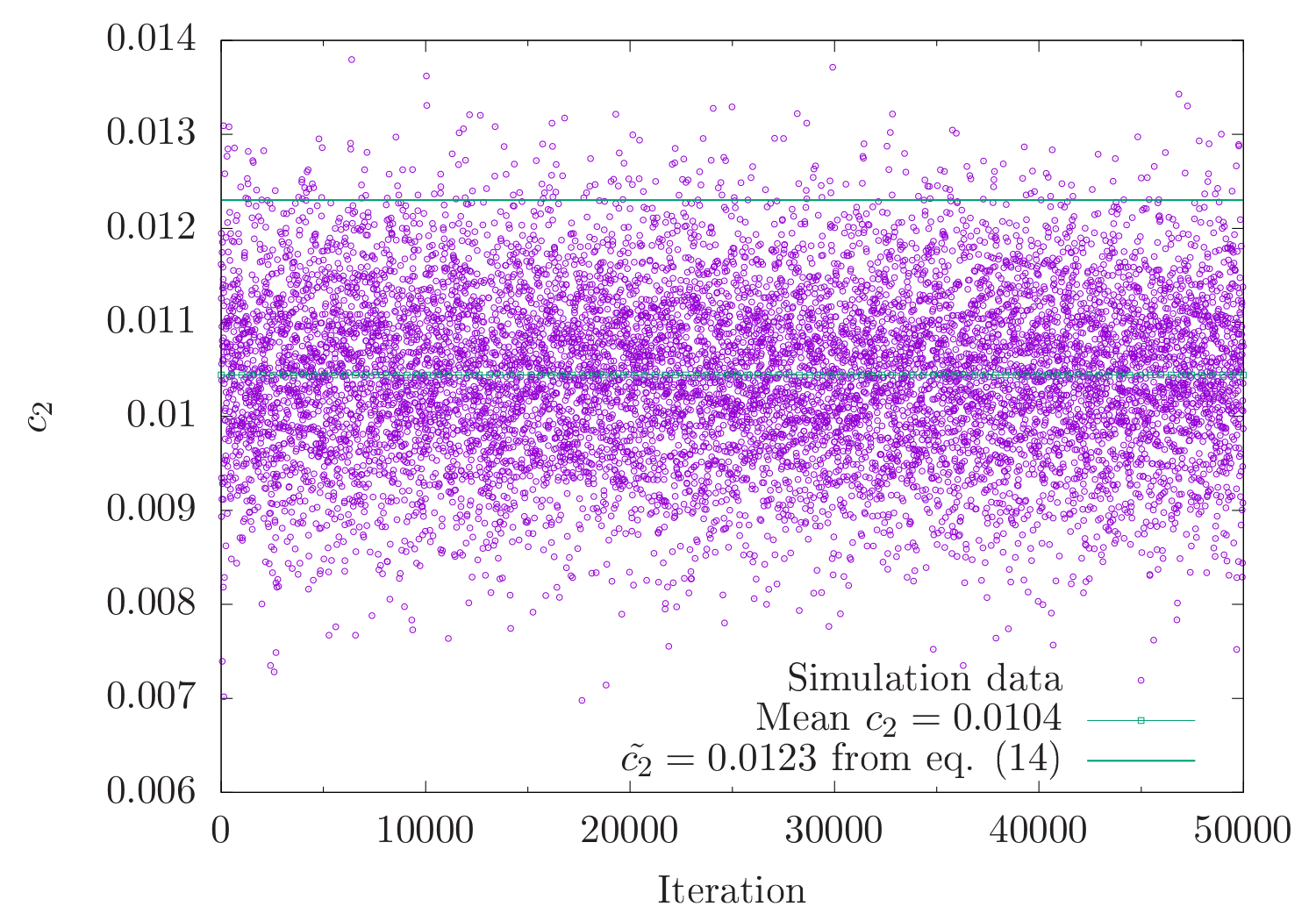

Supplement: Supplementary file 1 [file ECE3-10-1278-s001.tif]

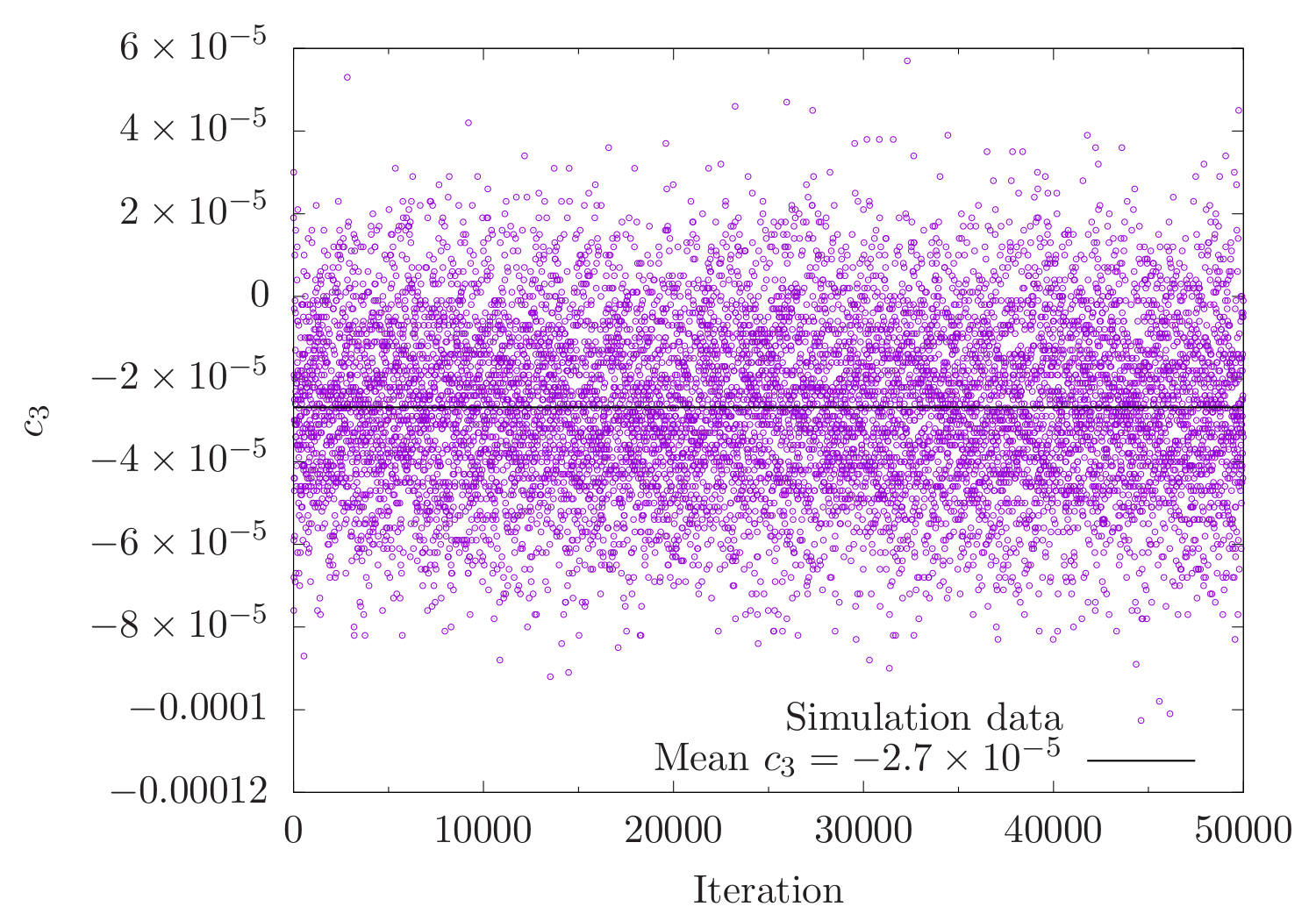

Supplement: Supplementary file 2 [file ECE3-10-1278-s002.tif]

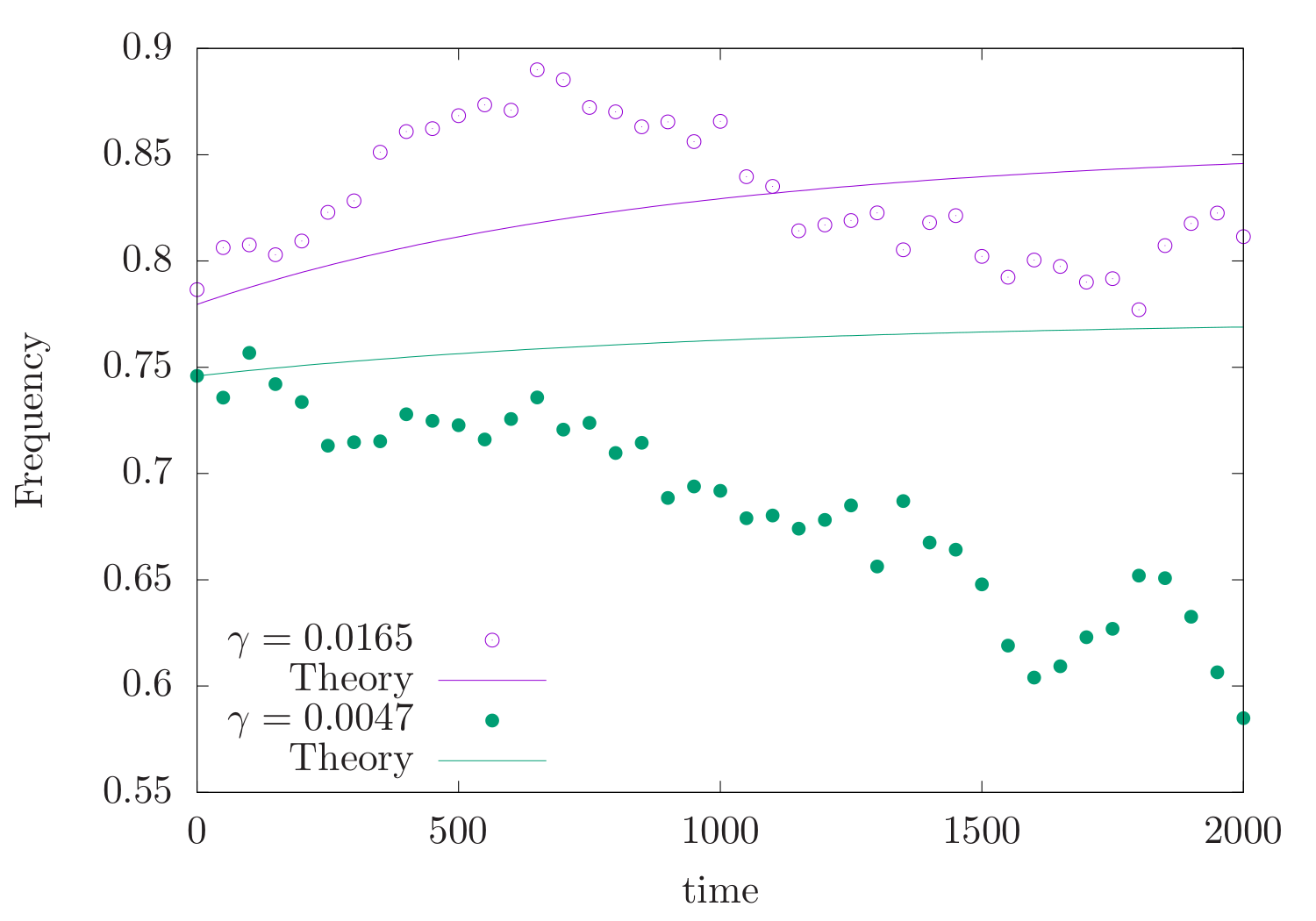

Supplement: Supplementary file 3 [file ECE3-10-1278-s003.tif]

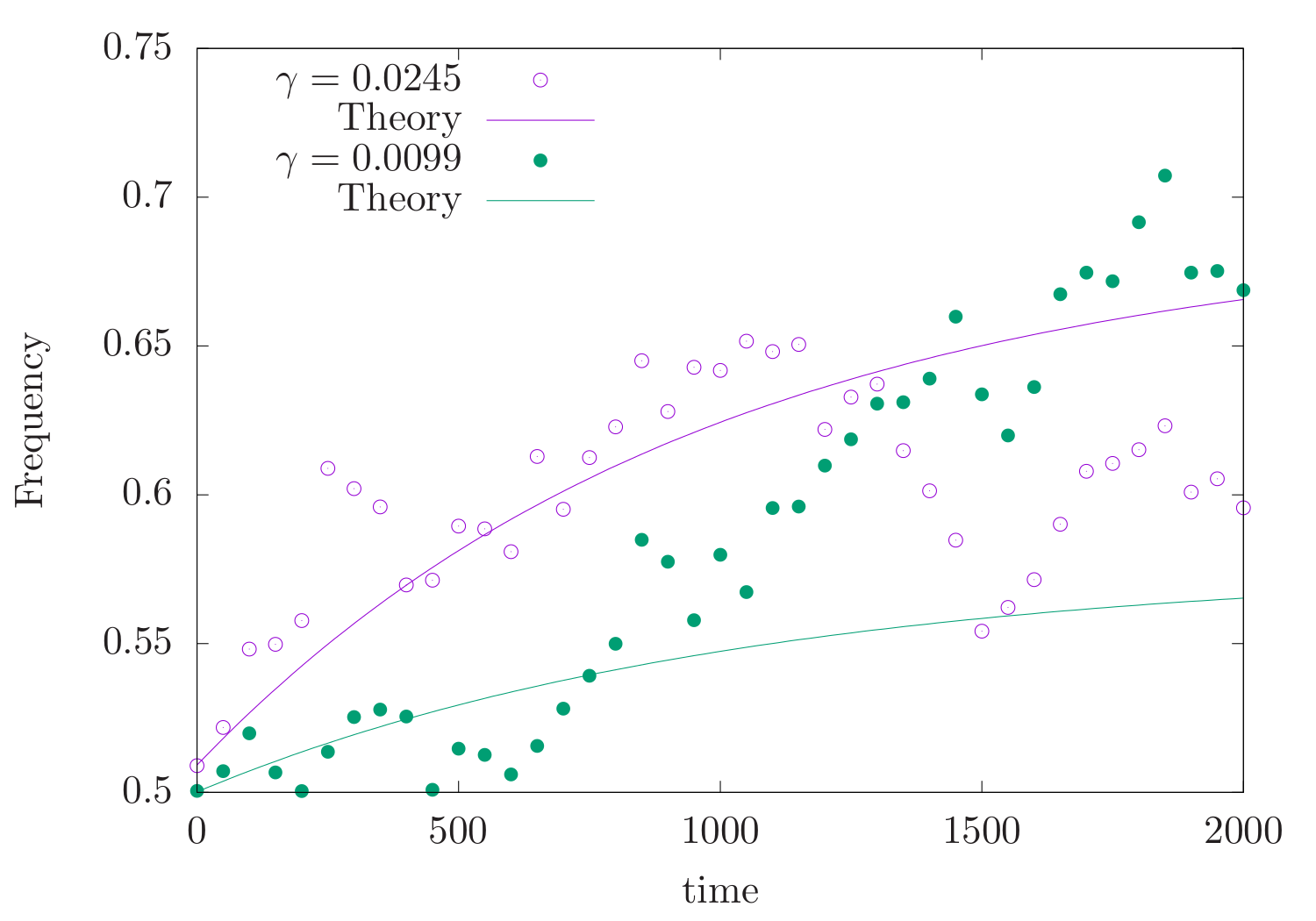

Supplement: Supplementary file 4 [file ECE3-10-1278-s004.tif]

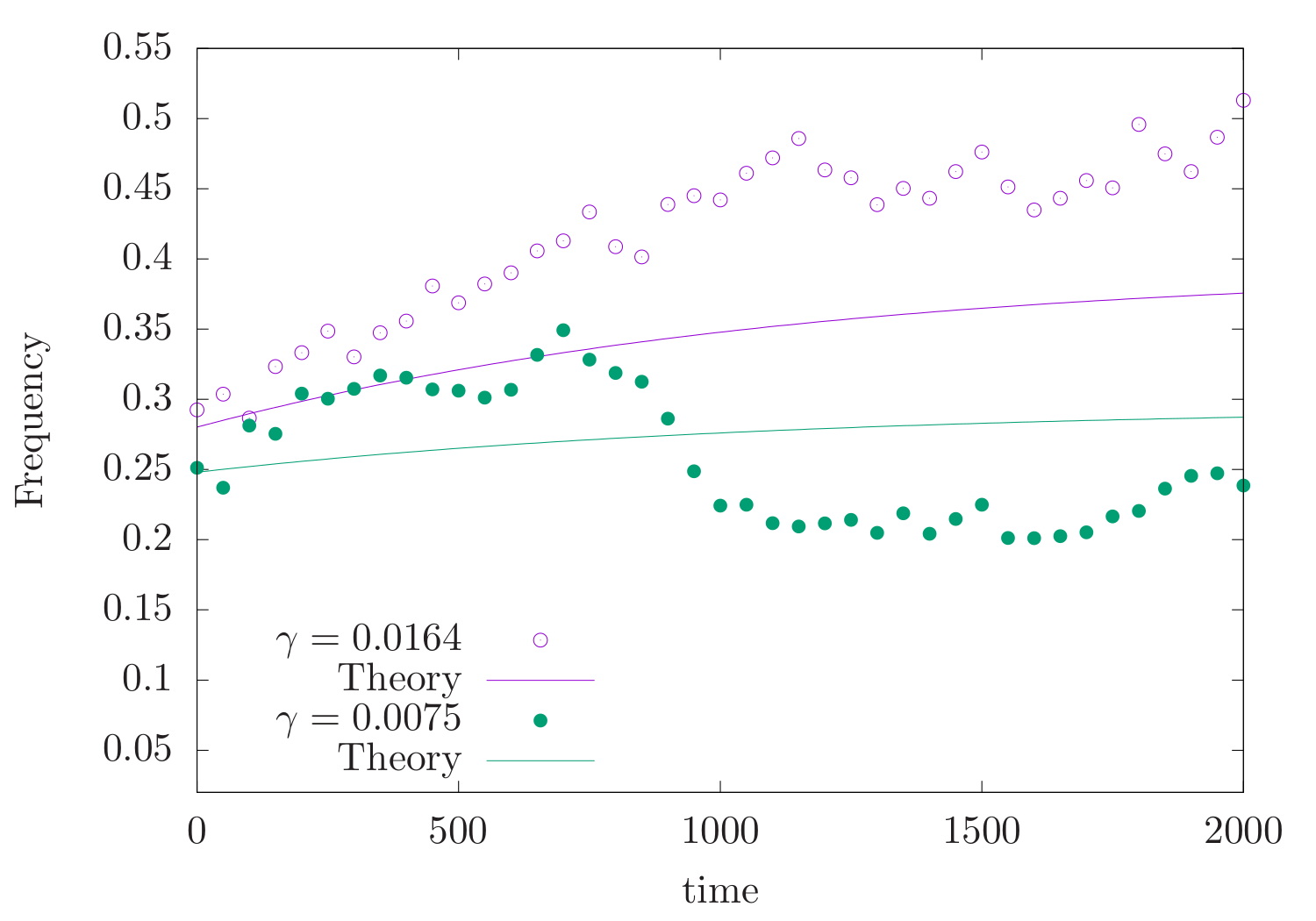

Supplement: Supplementary file 5 [file ECE3-10-1278-s005.tif]
